# Tartar Emetic Ointment in Chorea

**Published:** 1830

**Authors:** 


22. *Tartar Emetic Ointment in Chorea.* In the same Journal, Dr. Byrne of Baltimore, has reported two cases of chorea cured by frictions to the spine with tartar emetic ointment, as recommended by Dr. Jennen. In both, as soon as pustules made their appearance the disease subsided.

